# Peritoneal Expression of Membrane Complement Regulators Is Decreased in Peritoneal Dialysis Patients with Infected Peritonitis

**DOI:** 10.3390/ijms24119146

**Published:** 2023-05-23

**Authors:** Sosuke Fukui, Masashi Mizuno, Mitsuhiro Tawada, Yasuhiro Suzuki, Hiroshi Kojima, Yoshihisa Matsukawa, Masaki Imai, Hangsoo Kim, Hiroshi Kinashi, Makoto Mizutani, Kenichi Minoshima, Shoichi Maruyama, Yasuhiko Ito

**Affiliations:** 1 Department of Nephrology, Nagoya University Graduate School of Medicine, Nagoya 466-8550, Japan; 2 Department of Renal Replacement Therapy, Nagoya University Graduate School of Medicine, Nagoya 466-8550, Japan; 3 Department of Urology, Nagoya University Graduate School of Medicine, Nagoya 466-8550, Japan; 4 Department of Immunology, Nagoya City University, Nagoya 467-8601, Japan; 5 Department of Nephrology and Rheumatology, Aichi Medical University, Nagakute 480-1195, Japan; 6 Handa City Hospital, Handa 475-8599, Japan; 7 Daiyukai Daiichi Hospital, Ichinomiya 491-8551, Japan

**Keywords:** peritoneal dialysis, PD-related peritonitis, fungal infection, complement, membrane complement regulator

## Abstract

In peritoneal dialysis (PD) patients, fungi and *Pseudomonas aeruginosa* are considered important causative microorganisms for peritonitis with poor prognosis. Our objective was to explore expressions of membrane complement (C) regulators (CRegs) and tissue injuries in the peritoneum of patients with PD-related peritonitis, including fungal and *Pseudomonas aeruginosa* peritonitis. In peritoneal biopsy tissues obtained at PD catheter removal, we investigated the severity of peritonitis-associated peritoneal injuries and the expression of CRegs, CD46, CD55, and CD59 against peritoneal tissues without any episode of peritonitis. In addition, we evaluated peritoneal injuries among fungal and *Pseudomonas aeruginosa*-peritonitis (P1) and Gram-positive bacterial peritonitis (P2). We also observed deposition of C activation products such as activated C and C5b-9 and measured sC5b-9 in the PD fluid of patients. As a result, the severity of peritoneal injuries correlated inversely with the expression of peritoneal CRegs. Peritoneal CReg expression in peritonitis was significantly reduced compared to no peritonitis. Peritoneal injuries were more severe in P1 than in P2. CReg expression was further decreased and C5b-9 further increased in P1 than in P2. In conclusion, severe peritoneal injuries due to fungal and *Pseudomonas aeruginosa*-peritonitis decreased CReg expression and increased deposition of activated C3 and C5b-9 in the peritoneum, suggesting that peritonitis, particularly fungal and *Pseudomonas aeruginosa*-peritonitis, might induce susceptibility to further peritoneal injuries due to excessive C activation.

## 1. Introduction

Peritoneal dialysis (PD) is a renal replacement therapy (RRT) that is important for home health care. In Japan, as in many other countries, the population continues to age, with a consequent increase in the number of patients requiring RRT. Against this background of recent changes in society, the benefits of PD are making this RRT more and more feasible as a milder option for dialysis than hemodialysis. However, the risk of infectious peritonitis remains a critical problem for long-term PD therapy, as described in many reports [1,2,3]. When considering causative microorganisms for PD-related peritonitis, fungi and *Pseudomonas aeruginosa* (*P. aeruginosa*) are associated with particularly poor prognosis, with the possibility of lethal encapsulating peritoneal sclerosis (EPS) [4,5]. When fungal peritonitis is diagnosed, cessation of PD and catheter removal are still recommended and refractory peritonitis is common [6]. *P. aeruginosa* is also a common cause of refractory peritonitis and with proven infection, removal of the PD catheter is again recommended to preserve peritoneal tissue as stated in the 2016 and 2022 International Society for Peritoneal Dialysis (ISPD) guidelines [4,6,7].

Although the complement (C) system plays important roles in protecting the host from invading microorganisms and maintaining homeostasis [8], failure of the balance between activation and regulation is known to induce an excessive activation of the C system that contributes to various pathologies [8,9]. In our previous report, we used a peritonitis model of fungal infection involving severe and progressive peritoneal injuries driven by zymosan, a yeast cell membrane compound. Zymosan activates the C system through the alternative and lectin pathways [10]. Recently, we also showed that zymosan-induced peritonitis with methylglyoxal pretreatments could develop severe peritonitis and partially induce encapsulating changes in the gastrointestinal peritoneum. As known methods of preventing other tissue injuries [8], anti-C agents such as soluble C receptor 1, Crry-fusion protein, and C5a antisense peptide have been tested to prevent those peritoneal injuries in rat models [10,11]. In patients with PD-related peritonitis or newly started on PD, increases in C activation products such as sC5b-9 have the potential to predict prognosis, and increased C3a levels in PD fluid(PDF) predict the risk of developing eosinophilic peritonitis [12,13,14]. We have also focused on the roles of peritoneal membrane C regulators (CReg) using in vitro experiments and animal models [10,15,16]. We showed changes in the expression of CD55 (classified as a CReg) in human peritoneal mesothelium associated with peritoneal PD therapy, which were confirmed in another report [17,18]. However, little information is available regarding changes associated with the C system impacting the pathologies of PD-related peritonitis.

The present study investigated peritoneal tissues harvested from PD patients with and without peritonitis by peritoneal biopsy when the PD catheter was removed. We evaluated the distributions of CRegs and deposition of C activation products in peritoneal biopsy samples.

## 2. Results

### 2.1. Expressions of CRegs, CD46, CD55, and CD59 Are Decreased in Injured Peritoneum with Peritonitis

**Figure 1:**
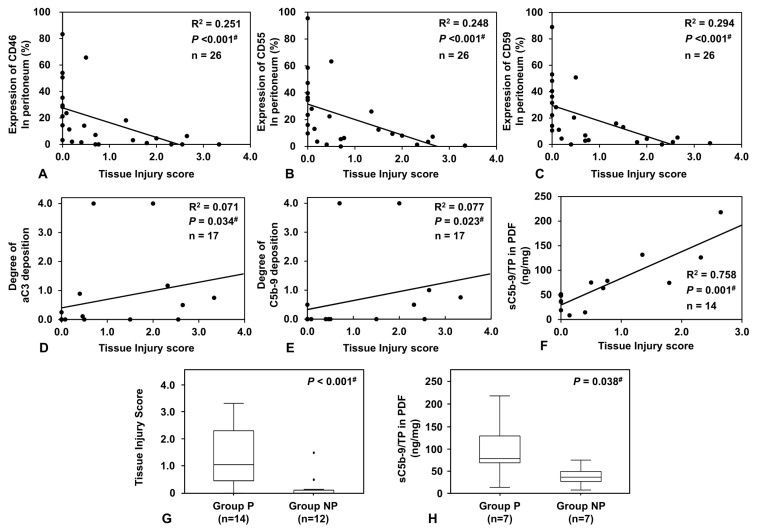
Correlations of expressions of membrane complement regulators (CRegs), CD46, CD55, and CD59, deposition of complement (C) activation products such as aC3 and C5b-9, and levels of sC5b-9 in peritoneal dialysis fluid (PDF) with tissue injury score and comparison of sC5-9 levels in PDF between Groups P and NP. Expressions of each of the CRegs CD46, CD55, and CD59 showed good inverse correlations with the severity score for peritoneal injuries ((**A**–**C**) respectively). In contrast, deposition of C activation products such as activated C3 (aC3) and C5b-9 correlated with the severity score of peritoneal tissue injures ((**D**,**E**) respectively). Adjusted PDF levels of sC5b-9 (sC5b-9/TP) also correlated with the severity score for peritoneal injures (**F**). Between Groups P and NP, the tissue injury score and PDF levels of sC5b-9/TP were significantly different ((**G**,**H**), respectively). Dots in the graph show outliers. Outliers were defined as values greater than the 75th percentile plus 1.5 times the interquartile range. ^#^, *p* < 0.05.

Peritoneal injuries were scored to estimate the severity of tissue injury. The tissue injury score was derived from the accumulation of inflammatory cells and fibrin exudation stained by phosphotungstic acid hematoxylin (PTAH) in peritoneal biopsy tissues. The tissue injury score showed a significant inverse correlation with the expression of CRegs CD46, CD55, and CD59 (Figure 1A–C). Deposition of activated C3 (aC3), which involved C3b, iC3b, C3c, and C5b-9 in the peritoneum, also correlated significantly with the tissue injury score (Figure 1D,E).

### 2.2. Comparison between Peritonitis (Group P) and Non-Peritonitis (Group NP) Patients

#### 2.2.1. Correlation between sC5b-9 Levels in PDF and Tissue Injury Score

Levels of sC5b-9 measured in PDF collected from tissue donors showed significant positive correlations with the tissue injury score in the peritoneum (Figure 1F). We divided the collected samples into two groups, from patients with peritonitis (Group P) or from patients with no history of peritonitis (Group NP). The tissue injury score and the adjusted levels of sC5b-9 in PD effluent were significantly higher in Group P than in Group NP (Figure 1G,H). The detailed rationale for how we determined groups is provided in the Materials and Methods section.

#### 2.2.2. Comparison of Blood Markers between Group P and Group NP

Leucocyte count and serum levels of C-reactive protein, both representing systemic markers of inflammation, were significantly increased in Group P compared to Group NP. In contrast, levels of albumin were significantly decreased in Group P. Age, sex, incidence of diabetes mellitus, PD history, peritoneal transport status, and PD adequacy did not differ significantly between groups. The details are shown in Table 1.

#### 2.2.3. Pathological Changes Are More Severe in Peritoneal Tissues with Peritonitis

**Figure 2:**
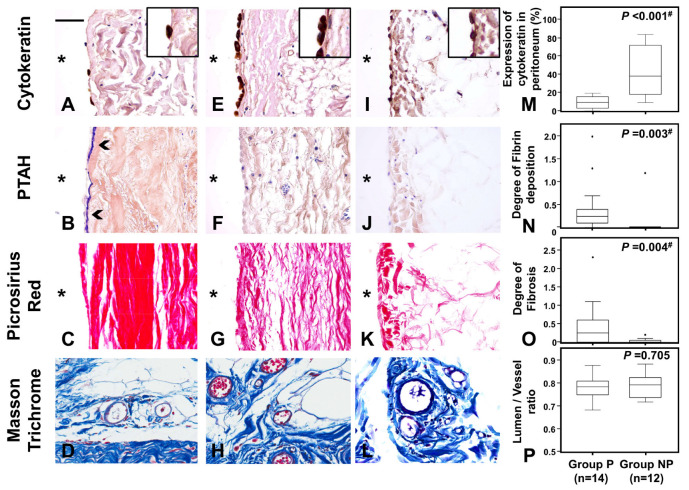
Fibrin exudation and fibrosis in the peritoneum between those with and without peritonitis. Frame sets (**A**,**E**,**I**), (**B**,**F**,**J**), (**C**,**G**,**K**), and (**D**,**H**,**L**) show the distributions of mesothelial cells stained by anti-cytokeratin, deposition of fibrin by phosphotungstic acid hematoxylin (PTAH) staining, distribution of fibrosis by picrosirius red staining, and vessels stained by Masson’s trichrome (MT), respectively. Frames (**A**–**D**) and (**E**–**H**) represent peritoneal tissues from patients with peritonitis (Group P) and patients without peritonitis (Group NP), respectively. As references, frames I–L showed control peritoneal tissues from a living renal transplantation donor (Control). Graphs (**M**–**P**) show results for proportions of mesothelial cells shown by anti-cytokeratin, scores of PTAH staining and picrosirius red staining, and lumen/vessel ratio reflecting the degree of vasculopathy under MT staining, respectively. Fibrin deposition (arrowhead) are shown in B. Original magnification of (**A**–**L**) is ×400. Right upper inserts in A and E show double the original magnification. A scale bar of 50 μm is shown in the left upper corner of frame A. * Peritoneal cavity side. Dots in the graph show outliers. Outliers were defined as values greater than the 75th percentile plus 1.5 times the interquartile range. ^#^, *p* < 0.05.

When we compared impairments of mesothelial cells, fibrin, and degree of fibrosis in the peritoneum between Groups P and NP, the numbers of cytokeratin-positive mesothelial cells along the peritoneum were clearly decreased in Group P compared with Group NP (Figure 2A,E,M), suggesting that peritoneal injury decreases mesothelial cell numbers in the peritoneum. Increased deposition of fibrin and degree of fibrosis were seen in the peritoneum with peritonitis (Group P), compared to the peritoneum without peritonitis (Group NP) (Figure 2B,C,F,G,N,O). Peritoneal vasculopathy, reported as a marker to predict progression to EPS, could not be found in most samples, so no significant differences were observed in the ratio of luminal diameter to vessel diameter (L/V ratio) values between groups (Figure 2D,H,P). Comparisons with previously reported levels [19] suggest that L/V ratios were similar to those of pre-PD patients.

**Figure 3:**
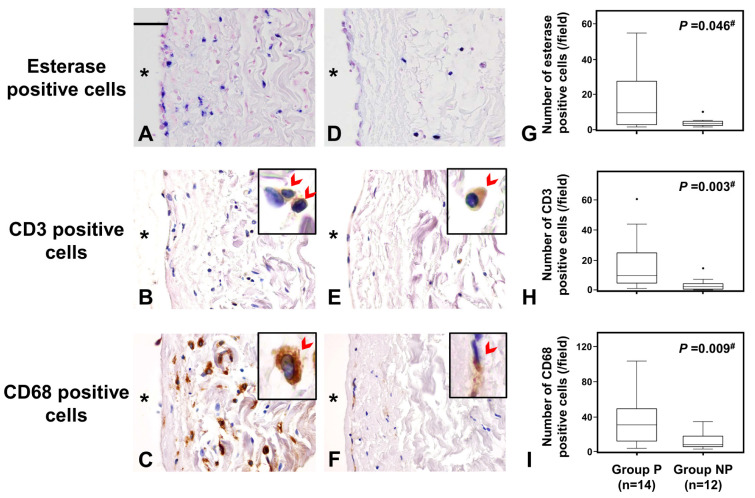
This figure shows the comparison of the accumulation of inflammatory cells in the peritoneum between those with peritonitis and those without. Frames and graphs show esterase-positive neutrophils (**A**,**D**,**G**), CD3-positive pan T-cells (**B**,**E**,**H**), and CD68-positive pan macrophages (**C**,**F**,**I**), respectively. Sets of photo frames (**A**–**C**) and (**D**–**F**) are peritoneal tissues of peritonitis in group P and of non-peritonitis in group NP, respectively. Accumulations of neutrophils, pan T-cells, and macrophages are observed in the peritoneum of group P significantly more than those of group NP. The original magnification of (**A**–**F**) was ×400 and a scale bar of 50 μm is shown in the left upper corner of frame A. * shows peritoneal cavity side. CD3/CD68—positive cells (arrowhead) are shown in right-upper corner of (**B**,**C**,**E**,**F**), which are magnified four times relative to the original photo. Dots in the graph show outliers. Outliers were defined as values greater than the 75th percentile plus 1.5 times the interquartile range. ^#^ *p* value < 0.05 is significance.

Comparison of peritoneal inflammatory cell infiltration between Groups P and NP showed that the accumulation of esterase-positive neutrophils, CD3-positive T cells, and CD68-positive macrophages was significantly greater in Group P than in Group NP (Figure 3).

#### 2.2.4. Expression of CRegs Is Decreased in Peritoneum with Peritonitis and Associated with Increased Complement Activation

**Figure 4:**
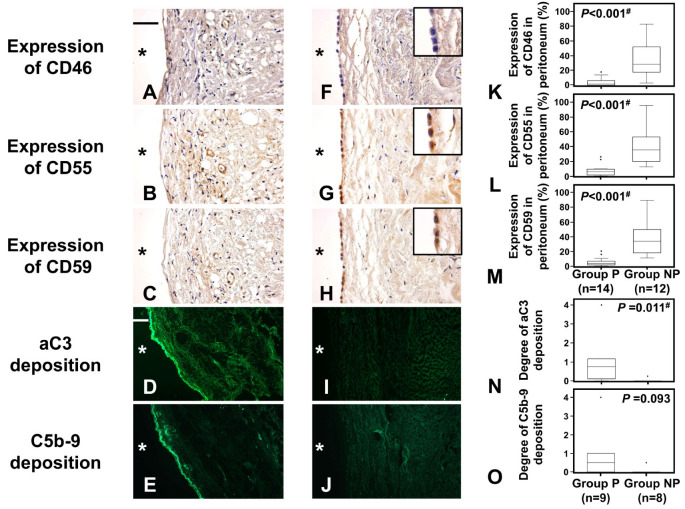
Expressions of CRegs CD46, CD55, and CD59 in those with and without peritonitis and depositions of complement (C) activation products. Frames (**A**–**E**) and (**F**–**J**) represent peritoneal tissues in Group P and Group NP, respectively. Frames (**A**,**F**), frames (**B**,**G**), frames (**C**,**H**), frames (**D**,**I**), and frames (**E**,**J**) show expressions of CD46, CD55, and CD59 and depositions of activated C3 (aC3) and C5b-9, respectively. Graphs (**K**–**O**) show results for expressions of CD46, CD55, and CD59 and deposition of aC3 and C5b-9, respectively. Original magnification of (**A**–**C**) and (**F**–**H**) is ×400, while that of (**D**,**E**,**I**,**J**) is ×100. Inserted frames in right upper corners of frames (**F**–**H**) are at double the original magnification. Scale bars of 50 μm and 200 μm are shown in the left upper corner of frames (**A**,**D**), respectively. * shows peritoneal cavity side. Dots in the graph show outliers. Outliers were defined as values greater than the 75th percentile plus 1.5 times the interquartile range. ^#^ *p* < 0.05.

Peritoneal expression of CRegs was compared between Groups P and NP. Expression of CD46, CD55, and CD59 was significantly decreased in Group P compared to Group NP (Figure 4A–C,F–H,K–M). In contrast, peritoneal deposition of aC3 and C5b-9 as the complement activation products was significantly increased in Group P compared to Group NP, suggesting that reduced CReg expression was associated with complement dysregulation in tissue (Figure 4D,E,I,J,N,O).

### 2.3. Comparison between Peritoneal Tissues with Peritonitis Due to Fungi and P. aeruginosa (Group P1) and Due to Gram-Positive Cocci (Group P2)

#### 2.3.1. Peritoneal Injuries Are More Severe in Peritonitis with Fungal and *P. aeruginosa* Infections

**Figure 5:**
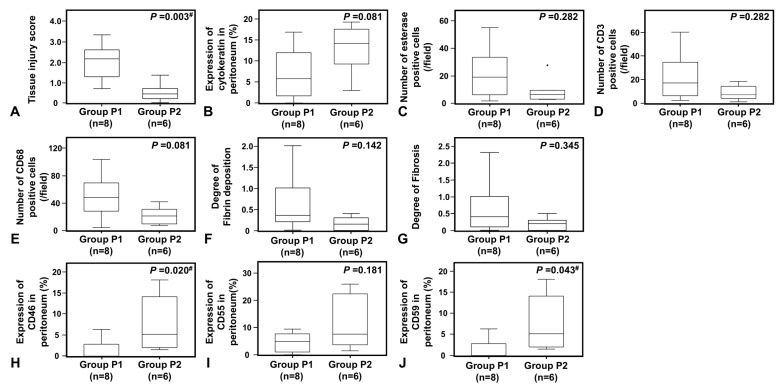
Pathological changes with accumulation of inflammatory cells of peritonitis and expression of membrane complement regulators (CRegs) caused by *Candida* spp. or *P. aeruginosa* (Group P1) and Gram-positive cocci (Group P2). Graphs (**A**,**B**) show the tissue injury scores and the proportion of cytokeratin-positive mesothelial cells in the peritoneum, respectively. Graph (**C**–**E**) shows the numbers of esterase-positive neutrophils, CD3-positive pan-T cells, and CD68-positive macrophages, respectively. Graphs (**F**,**G**) show the scores of fibrin deposition and fibrosis in the peritoneum, respectively. Graphs (**H**–**J**) show CRegs CD46, CD55, and CD59, respectively. Group P1 shows cases with fungal infection or *P. aeruginosa* and Group P2 shows cases with Gram-positive cocci. Dots in the graph show outliers. Outliers were defined as values greater than the 75th percentile plus 1.5 times the interquartile range. ^#^, *p* < 0.05.

The peritonitis group was subdivided into those with highly pathogenic fungal or *P. aeruginosa* infections (Group P1) and those with Gram-positive coccus infections (Group P2). Peritoneal injuries (tissue injury score) and expression of CRegs was compared between groups. Due to the small number of peritonitis biopsy samples caused by each microorganism, we gathered fungal and *P. aeruginosa* into Group P1 and pathologically compared the results with peritoneal biopsy samples caused by refractory/repeated peritonitis with Gram-positive cocci, which are usually considered to show better prognosis. The peritoneal injury score was significantly higher in Group P1 than in Group P2 (*p* < 0.005, Figure 5A).

When we compared the preservation of cytokeratin-positive cells between fungal and *P. aeruginosa*-associated peritonitis (Group P1) versus Gram-positive cocci-associated peritonitis (Group P2), a trend toward more profound cell loss was seen in Group P1, although this failed to reach significance (Figure 5B). In peritoneal tissues, inflammatory cell accumulation, fibrin exudation, and fibrosis were also increased in peritonitis associated with fungal and *P. aeruginosa* infections (P1) compared to those with Gram-positive cocci (P2), but the difference was not significant (Figure 5C–G).

#### 2.3.2. Expression of CRegs Was Significantly More Decreased with Fungal or *P. aeruginosa* Peritonitis Than with Gram-Positive Cocci Peritonitis

When expression of CRegs in the peritoneum was compared between Groups P1 and P2, expressions of CD46 and CD59 were significantly more decreased in Group P1 and expression of CD55 tended to be decreased, but not significantly, compared to Group P2 (Figure 5H–J).

#### 2.3.3. Depositions of aC3 and C5b-9 in Human Peritoneum by Causative Microorganisms

**Figure 6:**
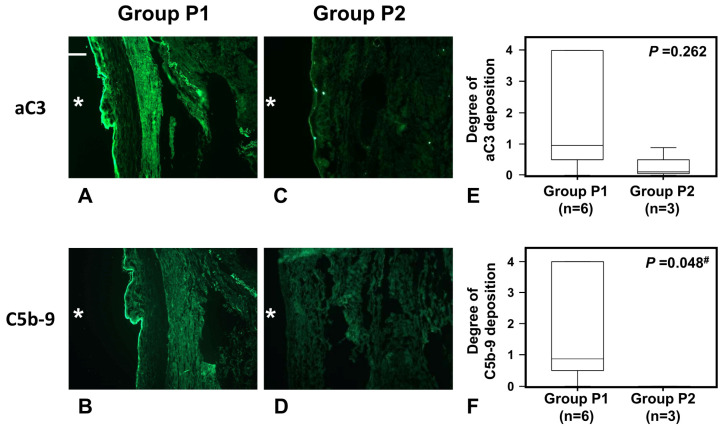
Deposition of C activation products aC3 and C5b-9 in fresh frozen tissues and in a typical biopsy tissue of peritoneum with peritonitis caused by fungus or P. aeruginosa (Group P1) and Gram-positive cocci (Group P2). Frame sets (**A**–**D**) show representative photos of deposition of aC3 and C5b-9 in peritoneum, respectively. Original magnification of (**A**–**D**) is ×100. A scale bar of 200 μm is shown in the left upper corner of frame A. Graphs (**E**,**F**) show degrees of aC3 and C5b-9 deposition, respectively. * Peritoneal cavity side. ^#^, *p* < 0.05.

Of note, the degree of C5b-9 deposition as a complement activation product was slightly but significantly higher in Group P1 peritoneum than in Group P2 peritoneum (*p* < 0.05) (Figure 6B,D,F), although the sample size was more limited for fresh frozen tissues than for paraffin-embedded tissues. Deposition of aC3 showed a similar trend to C9 deposition, but did not differ significantly between Groups P1 and P2 (Figure 6A,C,E).

Samples showing recognizable peritoneal surfaces were limited among fresh frozen samples and each evaluated sample is shown in Table 2 and Supplementary Figure S1.

## 3. Discussion

We have previously shown in animal models that infectious peritonitis, particularly fungal peritonitis, might develop severe and progressive inflammation in the peritoneum in association with excessive C activation [10,11,15,16,20]. We have also reported that impaired regulation of the C activation system might induce enormous fibrin exudation, similar to an early step in the development of EPS [16]. However, to date, scant pathological data has been available from PD patients to clarify the relationship between PD peritonitis-related peritoneal injuries and the C activation system.

The present study using human peritoneal biopsy samples showed a significant inverse correlation between the expression of CRegs and the peritoneal tissue injury score, and, in contrast, that depositions of aC3a and of C5b-9 correlated slightly but significantly with the tissue injury score in the peritoneum. Compared to patients without peritonitis (Group NP), peritoneal expression of CRegs was decreased in peritoneal biopsy samples from PD patients with peritonitis (Group P). Instead, depositions of C activation products were increased in Group P peritoneum, compared with Group NP.

Further analysis revealed that microscopic changes were more severe in Group P1 than in Group P2. Peritoneal expressions of CRegs CD46 and CD59 were also lower in Group P1 than in Group P2 and peritoneal C5b-9 deposition was more prominent in Group P1 than in Group P2. This suggested that fungal and *Pseudomonas* infections are both associated with poor prognosis, showing more severe peritoneal injuries with both diminished CRegs and greater activation of the C system compared to Group P2 as the reference peritonitis.

Peritoneal injuries may not only affect peritoneal function, but also the induction of EPS. Previous pathological examinations of non-peritonitis peritoneal biopsy samples from PD patients have found peritoneal thickening, detachment of mesothelial cells, accumulation of inflammatory cells, peritoneal fibrosis, and/or vasculopathy in the end-stage of renal disease patients on long-term PD, particularly for PD with low-pH PDF [19,21,22]. In addition to those peritoneal circumstances in PD patients, PD-related peritonitis has been another important reason underlying the development of peritoneal injuries and possibly progression to EPS, even with PD using pH-adjusted PDF [23]. Of note, in our study, no significant difference of vasculopathies and no significant progressions of vasculopathies could be observed between groups P and NP. Those findings might be found in PD patients mainly using pH-adjusted PDF [21,23]. The induction or production of various factors such as chemokines, growth factors, and inflammatory cells has been reported to cause and/or augment angiogenesis, lymphangiogenesis, mesenchymal-epithelial transition, fibrosis and, finally, the fall into EPS [24,25]. We focused on the C system as part of the system of innate immunity in the peritoneum, because dysfunction of the C system could be pathogenic in other tissues [8,9,26,27], and because results from animal models suggest that the C system may contribute to peritoneal injuries along with other factors and might facilitate progression to early-phase EPS [9,28]. In PD patients, we have previously reported that expression of CD55 correlated inversely with dialysate-to-plasma creatinine concentration ratio (D/P Cre) values in mesothelial cells and levels of sC5b-9 in PD effluent [18]. Expression of CD55 might also decrease with increasing severity of peritoneal injuries and increased D/P Cre under conditions of long-term acidic PDF according to in vitro assays [17]. The present study is the first to report evidence of altered CReg expressions, accompanied by deposition of C activation products and peritoneal injuries in PD patients with PD-related peritonitis using peritoneal biopsy samples. The present results appear to support our speculation that CReg dysfunction is a key mechanism accelerating C activation and facilitating severe peritoneal injuries in animal models of peritonitis [10,16].

**Figure 7:**
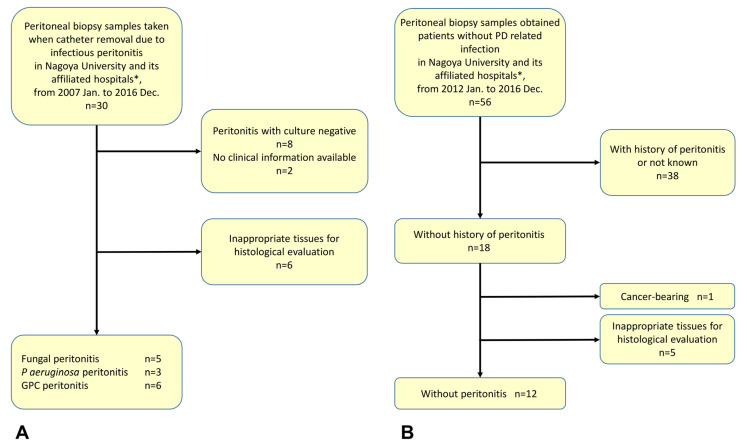
Analysis of peritoneal injuries in enrolled patients. Flow diagram (**A**) shows selection process to enroll patients with peritonitis (Group P). Flow diagram (**B**) shows selection process to enroll patients without peritonitis (Group NP). * Daiyukai Daiichi Hospital and Handa City Hospital; GPC, Gram-positive cocci; PD, peritoneal dialysis.

Some limitations of the present study need to be kept in mind. First, the sample size was small because opportunities for peritoneal biopsy were uncommon and the numbers of each microorganism were very small [1,2]. We therefore focused on three categories of microorganism: fungi; *P. aeruginosa*; and Gram-positive cocci. Only one sample at most was available for evaluation in patients with peritonitis caused by other microorganisms. A second limitation was the need to exclude other peritoneal biopsy samples with insufficient clinical data. Third, we had to eliminate all artificial changes during the procedure for harvesting peritoneal biopsy samples. We therefore finally evaluated limited peritoneal biopsy samples according to the process shown in Figure 7. Finally, a few patients temporally used acidic conventional PDF during the introduction period although pH-adjusted PDF was mainly used in the present study (see Table 2). Therefore, we could not completely remove the effects of acidic PDF. In addition, we could not completely distinguish between the acute phase and the chronic phase of the peritoneal pathological changes evaluated in this study.

In conclusion, the present study is the first to report that peritoneal injuries caused by peritonitis are accompanied by decreases in CRegs and might induce dysregulation of the C system with deposition of complement activation products in damaged peritoneum. Highly pathogenic microorganisms such as *Candida albicans* and *P. aeruginosa* might result in worse outcomes compared with other bacterial infections such as *Streptococci* and *Staphylococci*. From our results, severe peritoneal injuries could result in the loss of expression of CRegs in the peritoneum, and then this impaired CReg expression might induce unexpected C activation. Such C activation might then exert deleterious effects in the peritoneum and could further impair CReg expression in the peritoneum, similar to pathological findings in animal experiments [10]. Targeted anti-C therapy might thus become a treatment of choice to protect against peritoneal injuries during excessive C activation due to C dysregulation.

## 4. Materials and Methods

### 4.1. Antibodies and Agents

To investigate distributions of CRegs, an anti-human CD55 mouse monoclonal antibody (Ab) (clone 1C6), kindly donated by Dr. T. Fujita (Fukushima Medecal University, Fukushima, Japan), and an anti-human CD59 mouse monoclonal Ab (clone 1F5), were used as previously reported [18,29,30]. Monoclonal Ab (mAb) mouse anti-human CD46 (MEM 258) was purchased from BIO-RAD (Hercules, CA, USA). As an isotype control immunoglobulin (Ig)G1, mouse anti-rat CD46 (mAb MM.1; Hycult Biotech, Uden, The Netherlands), which is expressed only in male rat genital tissues, was used for immunohistochemical analyses [31]. Mouse anti-human cytokeratin (clone C-04) and rabbit anti-CD3 (cross-reacted with human) (clone SP7) were purchased from abcam (Kenbridge, UK), and mouse anti-human CD68 (clone PG-M1) was purchased from Dako (Glostrup, Denmark). The mAb anti-activated C3 (aC3, clone bH6; Hycult Biotech) detected depositions of C3b, iC3b, and C3c and the house-made polyclonal Ab (pcAb) anti-human C9, kindly gifted by B. Paul Morgan (Cardiff University, Cardiff, UK), was used to detect the deposition of C5b-9.

### 4.2. Peritoneal Biopsies from PD Patients

We selected 26 peritoneal biopsy samples collected during PD catheter removal in Nagoya University Hospital, Daiyukai Daiichi Hospital, or Handa City Hospital between January 2007 and December 2016 (Figure 7). Patient backgrounds and reasons for catheter removal are shown in Table 2. The present experiments were approved by the ethics committee from the Institute of Nagoya University Hospital (approval nos. #2005-0298, #2005-0299, #2005-0309, and #2013-0275), with agreement from committees from the other two institutes (Daiyukai Daiichi Hospital and Handa City Hospital). All participants provided written consent for the use of their laboratory data and biological materials.

First, we divided the collected samples into two groups for analysis, either those associated with peritonitis (Group P) or those without a history of peritonitis (Group NP). In the present study, the sample size for each microorganism was too small for statistical analysis of microorganism species-specific peritoneal injuries in Group P. Refractory peritonitis is common for both *P. aeruginosa* and fungal infections and prognosis is generally considered poor with a risk of developing EPS [4,5,6,7]. We therefore divided samples from Group P into two groups: P1, involving organisms associated with poor prognosis (5 samples of fungal peritonitis, 3 samples of *P. aeruginosa* peritonitis); and P2, involving organisms not associated with poor prognosis (6 samples of Gram-positive cocci) including 3 cases of refractory *Staphylococcus aureus* (*S. aureus*), one refractory case of *S. epidermidis*, one case of frequently recurrent peritonitis caused by *S. epidermidis* as the first causative microorganisms, *Micrococcus* spp. was sampled next, and finally *Staphylococcus* spp. was sampled over a period of approximately 4 months, plus one case of *β-Streptococcus* with *Enterococcus faecalis* (Group P2) (see detailed information in Table 2).

In PD-related peritonitis, catheter removal was performed immediately after the diagnosis of fungal infection and was categorized in the case of refractory/prolonged *P. aeruginosa* as a Gram-negative peritonitis and with prolonged *S. aureus* as a Gram-positive bacterial infection according to 2016 ISPD guidelines (6). Peritonitis cases were excluded if an obvious intrinsic cause of peritonitis was identified, including gastrointestinal perforations. As the reference, we used peritoneal tissue of a donor for renal transplantation.

### 4.3. Bacterial Cultures of PD Effluent to Identify Causative Microorganisms

For bacterial cultures to identify causative microorganisms, a combination of the two methods recommended in the 2010 ISPD guideline [32] was performed. Regarding patients presenting at the emergency room on bank holidays or at night, bacterial cultures were limited to the usage of culture bottles as per the recommendation from the 2016 and 2022 ISPD guidelines [6,7].

### 4.4. Tissue Preparation and Staining for Light and Immunofluorescent Microscopy

After collection, each peritoneal biopsy sample was divided, with half for fixation in 20% buffered formalin followed by paraffin embedding for light microscopy (LM) and half for embedding in tissue compound and snap freezing for immunofluorescent microscopy (IF). For small samples, LM was prioritized. To evaluate tissues under LM, paraffin-embedded tissues were sliced into 4-μm-thick sections. For IF observations, frozen tissues were sliced into 4-μm-thick sections using a cryostat, followed by acetone fixation at room temperature for 5 min.

### 4.5. Staining for Histological and Immunohistological Analyses

Biopsy sections were stained with hematoxylin and eosin (HE) to observe general findings of peritoneal tissue injuries, with PTAH reagent to detect fibrin formation, and with picrosirius red to evaluate fibrosis under LM, as described [11,33]. Peritoneal vasculopathy was evaluated using Masson’s trichrome (MT) with staining as previously reported [19,33]. Accumulated neutrophils in the peritoneum were identified using the Fast Blue Salt esterase reaction method [34]. Briefly, deparaffinized sections were incubated in chloroacetate solution (5 mg naphthol AS-D in 1 mL of N,N-dimethylformamide mixed with 25 mg fast blue BB salt in 40 mL of PBS) overnight at 4 °C in the dark. After rinsing, slides were stained with Nuclear Fast Red counterstain (Vector Laboratories, Burlingame, CA, USA) to counterstain nuclei.

To observe mesothelial cells and macrophages and study expressions of CD46, CD55, and CD59 in peritoneal samples as in our previous report [18], deparaffinized thin sections were incubated with mouse mAb C-04, mouse mAb PG-M1, mouse mAb MEM 258, mouse mAb 1C6, or mouse mAb 1F5, followed by pcAb goat anti-mouse IgG antibody and horseradish peroxidase-labeled polymer (Histofine^®^ Simple Stain Max-PO (M); Nichirei Biosciences, Tokyo, Japan). To identify T cells, rabbit mAb SP7 was used, followed by polyclonal goat anti-rabbit IgG antibody and horseradish peroxidase-labeled polymer (Histofine^®^ Simple Stain Max-PO (R); Nichirei Biosciences). Enzyme activity was detected using a 3,3′-diaminobenzidine tetrahydrochloride liquid system. Counterstaining was performed with hematoxylin. Before incubation of mAbs C-04, PG-M1, and SP-7, antigen retrieval was performed in deparaffinized sections as per our previous report [33].

To detect deposition of activated C3 (aC3) and C5b-9, monoclonal mouse anti-human aC3 to detect C3b, iC3b, and C3c (clone bH6) and house-made monoclonal mouse anti-human C9 were used, respectively. Secondary Abs comprising fluorescein isothiocyanate (FITC)-labeled goat anti-mouse IgG was purchased from Jackson ImmunoResearch (Westgrove, PA, USA).

### 4.6. Evaluation of Severity of Peritoneal Tissue Injuries, Fibrin Deposition, Peritoneal Fibrosis, and Vasculopathy

To analyze peritoneal tissue damage, a tissue injury score was calculated by summing scores from severity scales measuring two factors: (a) accumulation of inflammatory cells (scored as: 0, 0–50 cells per high-power field (HPF; ×400); 1, 51~100 cells/HPF; 2, 101~150 cells/HPF; 3, 151~200 cells/HPF; and 4, >201 cells/HPF); and (b) severity of fibrin deposition (scored as 0, negative; 1, minimal; 2, mild; 3, moderate; and 4, severe) under ×400 magnification. Peritoneal pathological finding of each typical score is shown in Supplementary Figure S2.

To evaluate chronic tissue damage, severity of fibrosis was scored as 0–4 for extent of picrosirius red-positive area in up to 30 random points for each section under ×200 magnification.

Vasculopathy was evaluated as L/V ratio in postcapillary venules with external diameters of 25–50 μm, in accordance with previous reports [19]. The mean L/V ratio of ten randomly selected vessels in each biopsy tissue was calculated, except for one sample in which only six vessels were found; in that case, the mean value of all six vessels was used.

### 4.7. Analysis of Mesothelial Cells in the Peritoneum and Expression of CRegs

For evaluation of cytokeratin-positive mesothelial cell expression of CRegs CD46, CD55, and CD59, we measured the positive staining length and full length of the peritoneal surface in each field under ×200 magnification, then calculated the positive area as:

Area of positive expression of cytokeratin, CD46, CD55, or CD59 (%) = (length of area staining positive for cytokeratin, CD46, CD55, or CD59 along peritoneal surface)/(full length of peritoneal surface) × 100(1)

The mean value of all fields for each specimen was calculated because specimen size was variable and small.

### 4.8. Analysis of Accumulation of Inflammatory Cells in Peritoneum

To investigate the accumulation of inflammatory cells in peritoneal tissues, all fields of each specimen were evaluated, because specimen size was variable and small. The average number of esterase-positive neutrophils, CD68-positive macrophages, and CD3-positive T cells per field was calculated for each sample under ×400 magnification.

### 4.9. Measurements of Total Protein and Soluble C5b-9 (sC5b-9) in PDF

To measure levels of sC5b-9 in PDF, the MicroVueTM sC5b-9 Plus EIA kit (Quidel Co., San Diego, CA, USA) was used according to the instructions from the manufacturer. Total protein in PDF was measured by BCA protein assay reagent (Thermo Fisher Scientific, Waltham, MA, USA). For each PDF, the level of sC5b-9 was adjusted for the total protein according to our previous report [13].

### 4.10. Statistical Analysis

Data are summarized as median and interquartile range for continuous variables and number and percentage for categorical variables. Continuous and categorical data were compared between two groups using the Mann-Whitney U test or Fisher’s exact test, respectively. To examine correlations between tissue injury score and deposition of CRegs or C activation products, Spearman’s rank correlation coefficients were calculated. A two-sided *p*-value < 0.05 was considered significant. Statistical analyses were carried out using IBM SPSS Statistics version 28.0 (International Business Machines Corp, Armonk, NY, USA).

## Figures and Tables

**Table 1:** Comparison of background characteristics between patients in Groups P and NP.

	Group P	Group NP	*p*-Value ^1^
Number of cases	14	12	
Age (years)	61.5 (57.3, 76.0)	64.0 (56.0, 70.0)	0.980
Male/Female	12 (85.7)/2 (14.3)	9 (75.0)/3 (25.0)	0.636
DM ^2^/non-DM	8 (57.1)/6 (42.9)	8 (66.7)/4 (33.3)	0.297
PD ^3^ history (months)	41.5 (20.3, 53.5)	60.0 (11, 103)	0.701
Serum albumin level (g/dL)	3.0 (2.7, 3.4)	3.7 (3.3, 4.0)	0.005 ^#^
Blood urea nitrogen level (mg/dL)	46.0 (29.9, 72.4)	51.0 (29.8, 66.2)	0.799
Serum creatinine level (mg/dL)	8.13 (7.03, 9.21)	9.76 (7.10, 11.16)	0.205
Serum C-reactive protein level (mg/dL)	4.67 (1.91, 11.33)	0.20 (0.05, 0.41)	<0.001 ^#^
Blood white blood cell count (/µL)	8035 (6385, 12,800)	6200 (4800, 8700)	0.039 ^#^
Blood hemoglobin level (g/dL)	10.9 (9.5, 11.6)	11.2 (11.0, 13.0)	0.137
Serum Beta-2-microgroblin (µg/mL)	24.6 (22.8, 42.8)	23.5(21.9, 41.1)	0.512
Adequacy for PD ^3^ (Kt/V)	1.71 (1.54, 1.90)	1.74 (1.38, 1.94)	0.600
Dialysate-to-plasma creatinine concentration ratio (D/P Cr) as a peritoneal transport status	0.68 (0.57, 0.75)	0.65 (0.59, 0.77)	0.786

Data are shown as median (interquartile range) or number (%). In group P, Laboratory data was collected from medical records when peritonitis occurred except for values of Kt/V and D/P Cr. Values of Kt/V and D/P Cr were before the incidence of peritonitis. ^1^ Mann-Whitney U test for continuous data, Fisher’s exact test for categorical data. ^2^ Diabetes mellitus. ^3^ Peritoneal dialysis. ^#^ *p* < 0.05 is significant.

**Table 2:** Demographic data for each patient.

Case Number	Cause of Withdrawal	Microorganism	Duration from Infection to Catheter Removal (Days)	Age	Sex	PD history until PD Catheter Removal (Months)	LM	IF	PDF	β2-MG (μg/mL)	Kt/V	D/P Cr	PD Solution
1	Peritonitis	*Candida species*	9	77	M	48	A	A	A	46	1.75	0.60	Glu ^1,2^
2	Peritonitis	*C. albicans*	14	58	M	21	A	A	A	16.4	1.89	0.66	Glu ^1^
3	Peritonitis	*C. albicans*	9	73	M	89	A	A	A	27.5	2.32	0.47	Glu^1^
4	Peritonitis	*C. albicans*	7	57	M	27	A	A	N/A	24.6	1.66	0.69	Glu ^1^
5	Peritonitis	*Candida species*	16	61	M	20	A	N/A	A	24.2	1.54	0.56	Glu ^1^
6	Peritonitis	*P. aeruginosa*	15	73	M	12	A	A	N/A	20.7	N/A	0.75	Glu ^1^
7	Peritonitis	*P. aeruginosa*	9	62	M	49	A	N/A	A	23.8	1.55	0/77	Glu ^1^
8	Peritonitis	*P. aeruginosa*	10	40	F	3	A	A	N/A	N/A	N/A	N/A	Glu ^1,2^/ico
9	Peritonitis	*S. aureus (MRSA)*	8	84	M	55	A	N/A	A	22.8	N/A	0.61	Glu ^1^
10	Peritonitis	*Β streptococcus E. faecalis*	37	80	M	16	A	N/A	N/A	N/A	N/A	N/A	Glu ^1^
11	Peritonitis	*Staphylococcus species*	127	66	F	42	A	A	N/A	34.7	N/A	0.55	Glu ^1^
12	Peritonitis	*S. aureus(MRSA)*	10	55	M	29	A	N/A	A	N/A	N/A	0.74	Glu ^1^/ico
13	Peritonitis	*S. aureus(MSSA)*	3	32	M	41	A	A	A	42.8	2.06	0.70	Glu ^1,2^
14	Peritonitis	*S. epidermidis*	15	60	M	69	A	A	N/A	47.9	2.00	0.85	Glu ^1^
15	To prevent EPS	-	-	64	F	119	A	A	A	22.8	2.58	0.59	Glu ^1,2^/ico
16	Over hydration	-	-	57	M	55	A	N/A	A	45.5	1.94	0.56	Glu ^1^/ico
17	Ultrafiltration failure	-	-	72	M	93	A	N/A	A	41	1.74	0.51	Glu ^1^/ico
18	Social problem	-	-	70	M	48	A	N/A	A	23.2	1.41	0.88	Glu ^1^/ico
19	To prevent EPS	-	-	43	M	103	A	A	A	22.7	1.97	0.63	Glu ^1^/ico
20	To prevent EPS	-	-	69	M	106	A	N/A	A	11.7	1.93	0.63	Glu ^1^/ico
21	Over hydration	-	-	73	M	9	A	A	A	23.8	1.21	0.68	Glu ^1^/ico
22	Ultrafiltration failure	-	-	56	M	11	A	A	N/A	19.3	1.86	0.75	Glu ^1^/ico
23	Ultrafiltration failure	-	-	40	M	10	A	A	N/A	N/A	1.22	N/A	Glu ^1^/ico
24	To prevent EPS	-	-	75	F	72	A	A	N/A	41.3	1.78	0.84	Glu ^1^/ico
25	Social problem	-	-	64	M	2	A	A	N/A	N/A	N/A	0.77	Glu
26	To prevent EPS	-	-	66	F	60	A	A	N/A	38.2	1.54	0.65	Glu ^1^/ico

^1^ The patient used pH adjusted glucose-containing solution. ^2^ The patient used conventional (acidic) PD solution for initial years after PD induction. PD, peritoneal dialysis; LM, sample for light microscopy; IF, fresh frozen sample for immunohistochemistry; PDF, peritoneal dialysis fluid sample; β2-MG, Beta-2-microgloblin; D/P Cr, dialysate-to-plasma creatinine concentration ratio; A, available; N/A, not available; Glu, Glucose-containing solutions; ico, icodextrin-containing solutions; C., Candida; P., Pseudomonas; S., Staphylococcus; MRSA, methicillin-resistant *S. aureus*; MSSA, methicillin-sensitive *S. aureus*; E, Enterococcus.

## Data Availability

The data presented in this study are available on reasonable request from the corresponding author. The data are not publicly available due to the necessity of obtaining individual agreement from the Ethics Committee at the Institute of Nagoya University Hospital.

## Supplementary Materials

The following supporting information can be downloaded at: https://www.mdpi.com/article/10.3390/ijms24119146/s1.
