# Supplementary material for: Peritoneal Expression of Membrane Complement Regulators Is Decreased in Peritoneal Dialysis Patients with Infected Peritonitis
Source: Int J Mol Sci. 2023 May 23;24(11):9146. doi: 10.3390/ijms24119146 (PMC10252763; doi:10.3390/ijms24119146)
Supplement: Supplementary file 1 [file ijms-24-09146-s001.zip › ijms-2390537-supplementary.pdf]

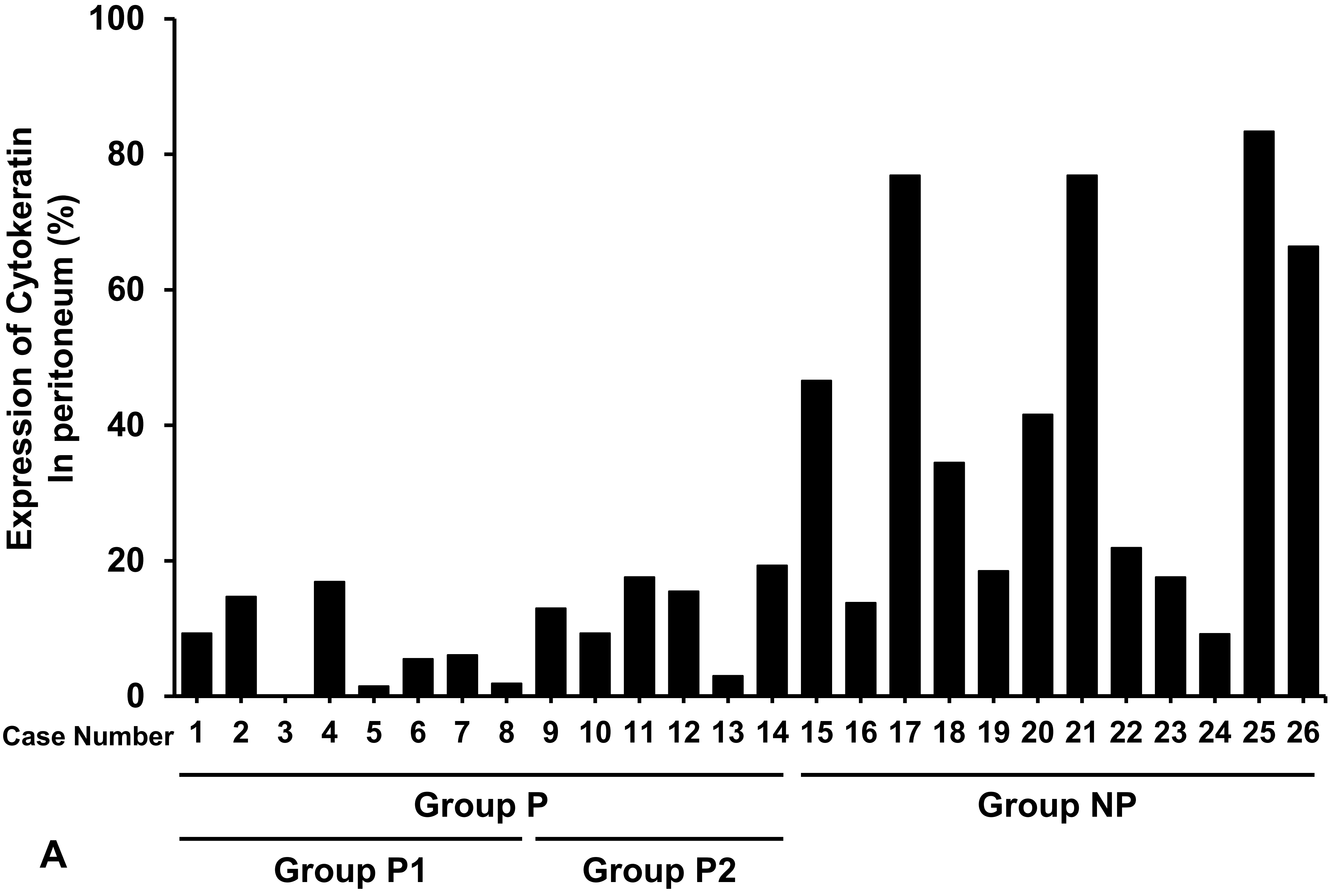

100
80
60
40
20
0
Expression of Cytokeratin
In peritoneum (%)
Case Number
1 2 3 4 5 6 7 8 9 10 11 12 13 14 15 16 17 18 19 20 21 22 23 24 25 26
Group P
Group NP
A
Group P1
Group P2

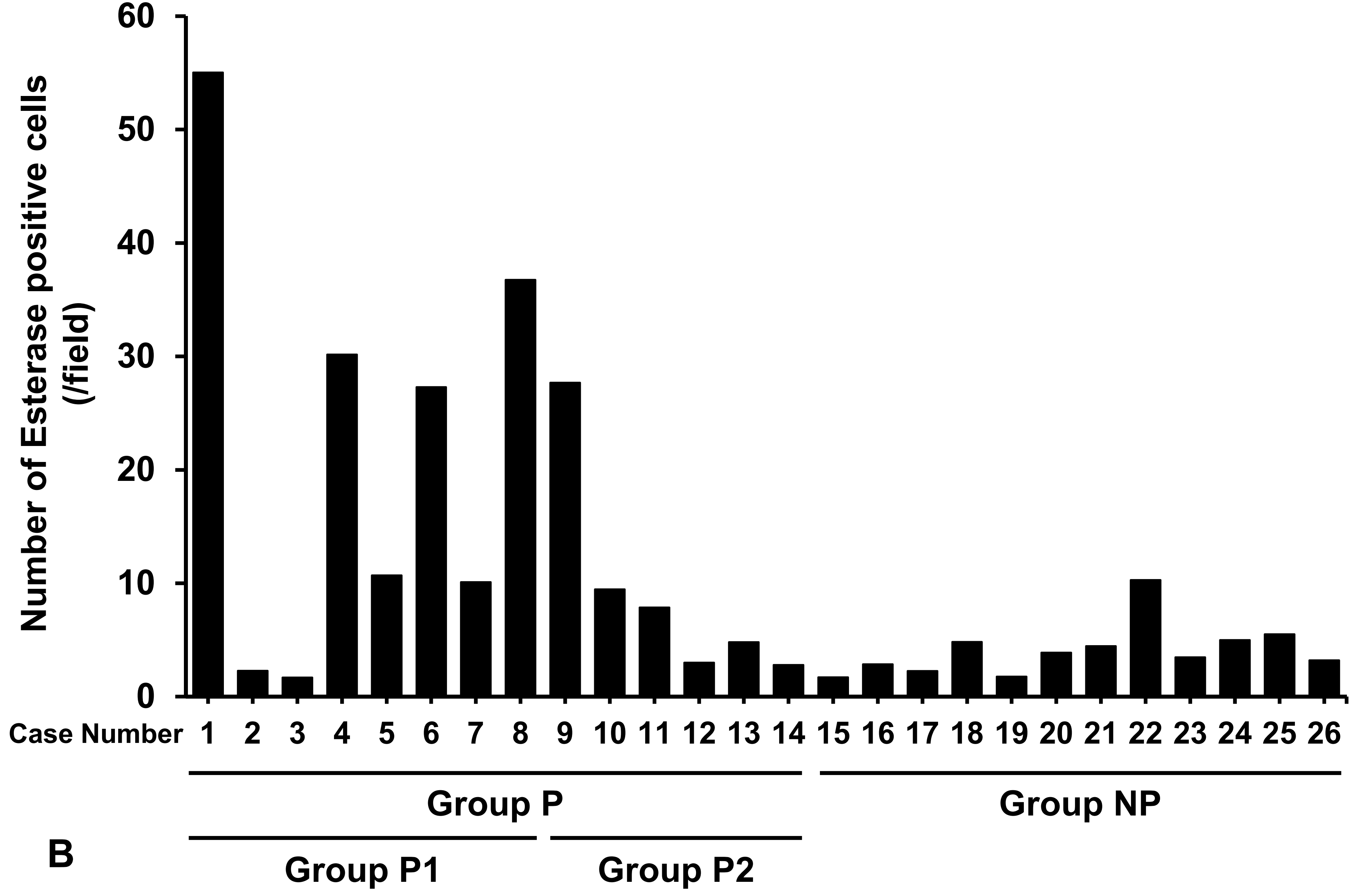

Number of Esterase positive cells (/field)
60
50
40
30
20
10
0
Case Number
1 2 3 4 5 6 7 8 9 10 11 12 13 14 15 16 17 18 19 20 21 22 23 24 25 26
Group P
Group NP
Group P1
Group P2
B

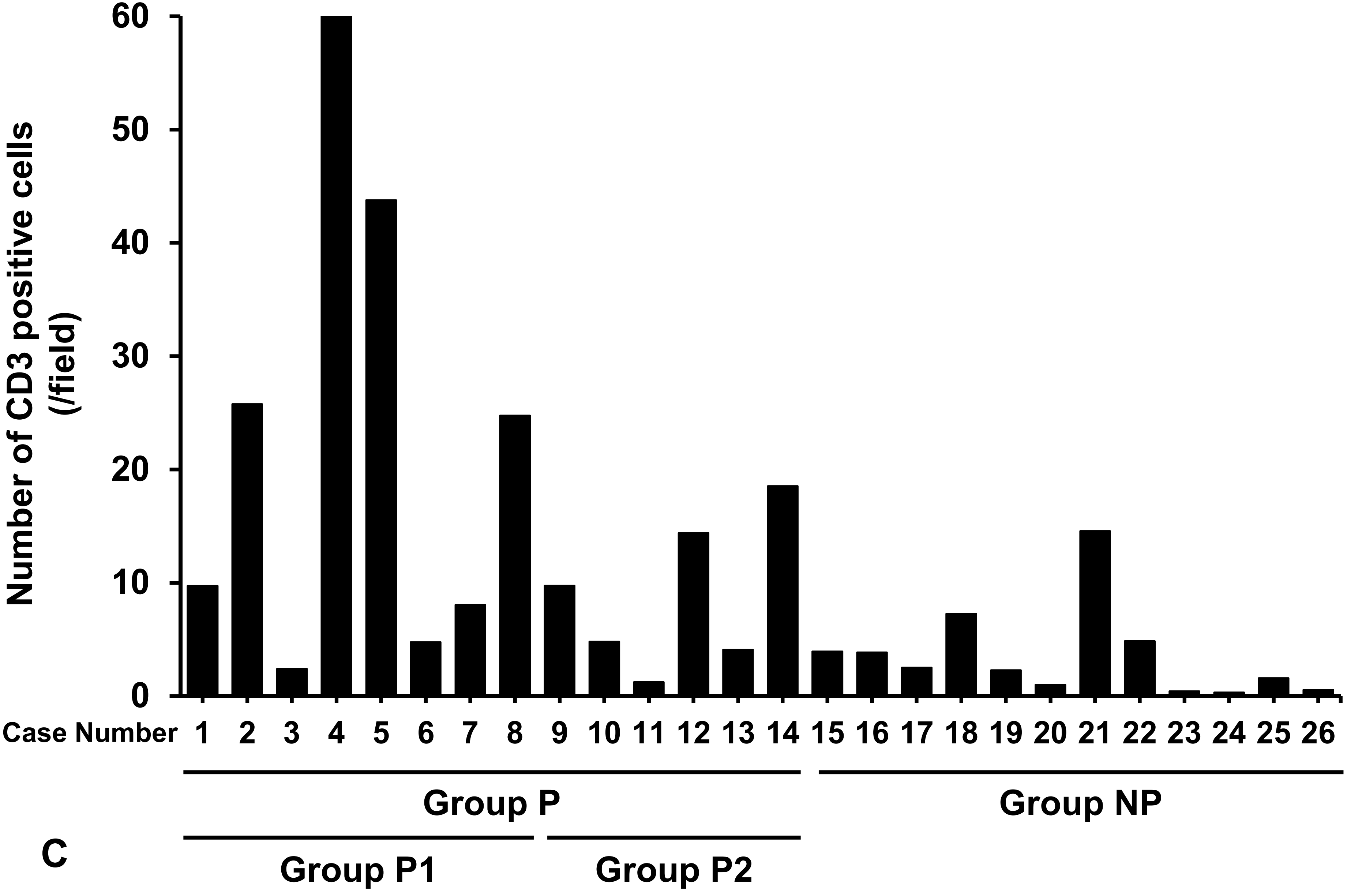

Number of CD3 positive cells (/field)
60
50
40
30
20
10
0
Case Number 1 2 3 4 5 6 7 8 9 10 11 12 13 14 15 16 17 18 19 20 21 22 23 24 25 26
Group P
Group NP
Group P1
Group P2

C

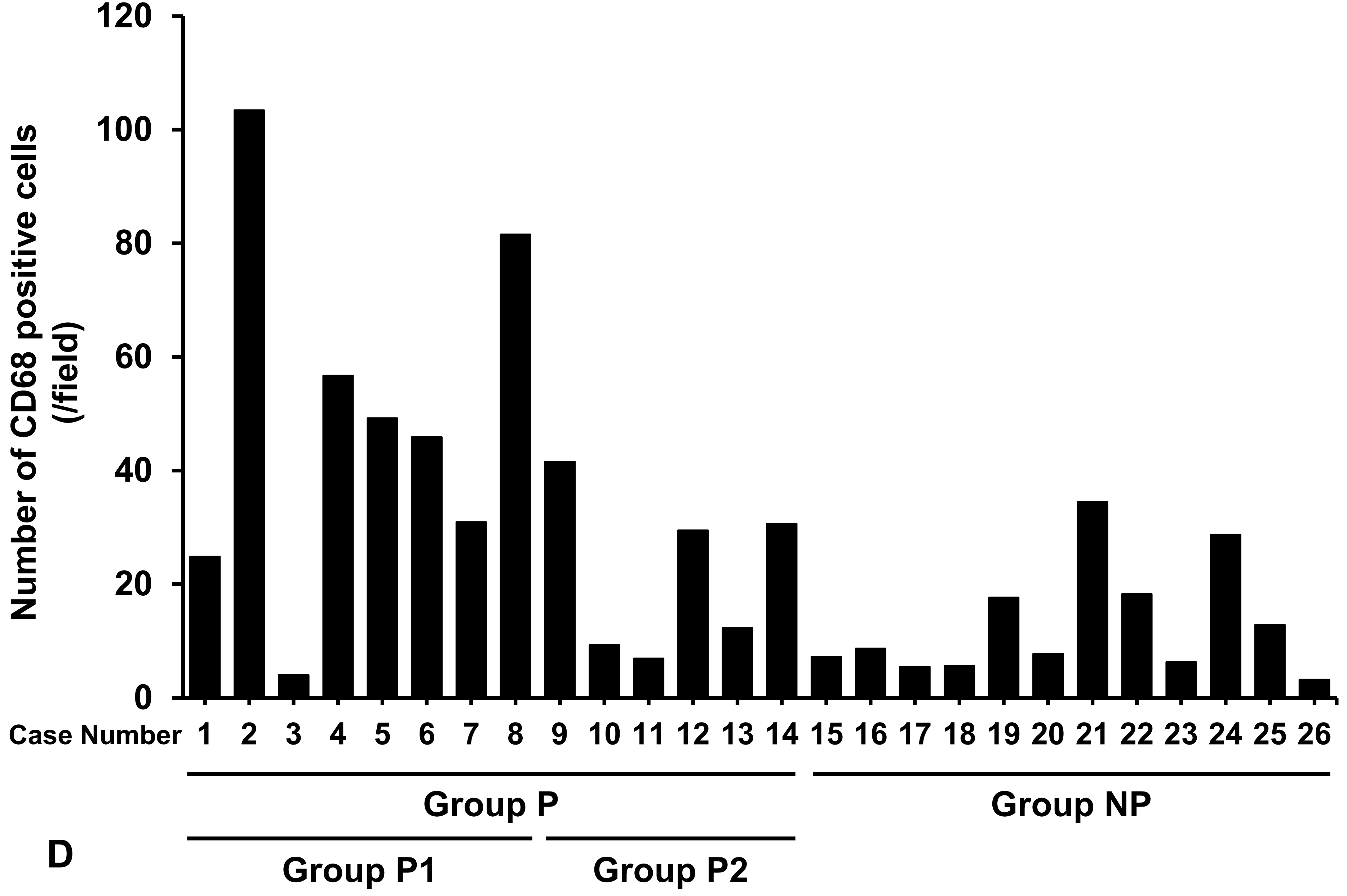

Number of CD68 positive cells (/field)
120
100
80
60
40
20
0
Case Number 1 2 3 4 5 6 7 8 9 10 11 12 13 14 15 16 17 18 19 20 21 22 23 24 25 26
Group P
Group NP
Group P1
Group P2

D

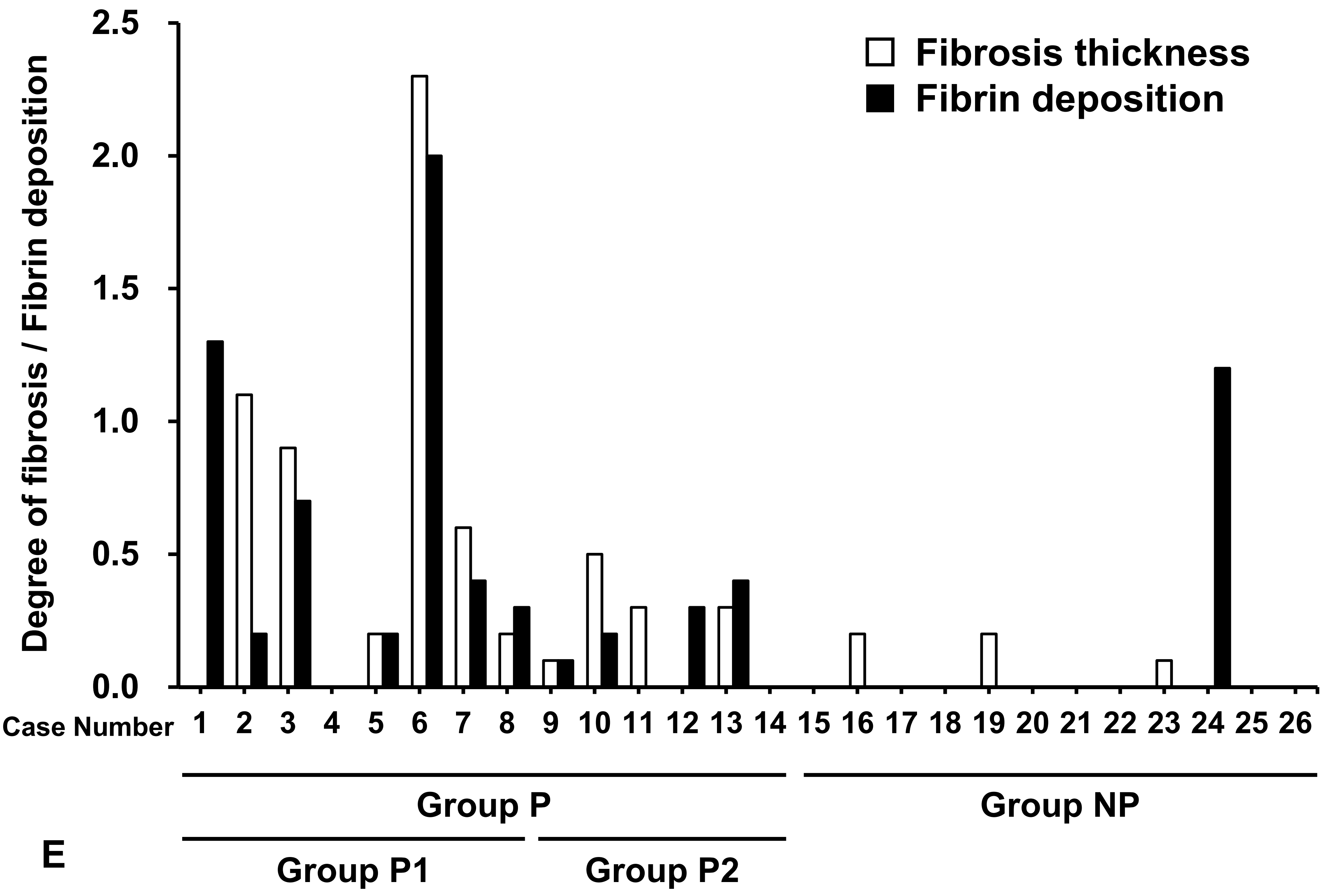

Fibrosis thickness
Fibrin deposition
Degree of fibrosis / Fibrin deposition
2.5
2.0
1.5
1.0
0.5
0.0
Case Number
1 2 3 4 5 6 7 8 9 10 11 12 13 14 15 16 17 18 19 20 21 22 23 24 25 26
Group P
Group NP
Group P1
Group P2

E

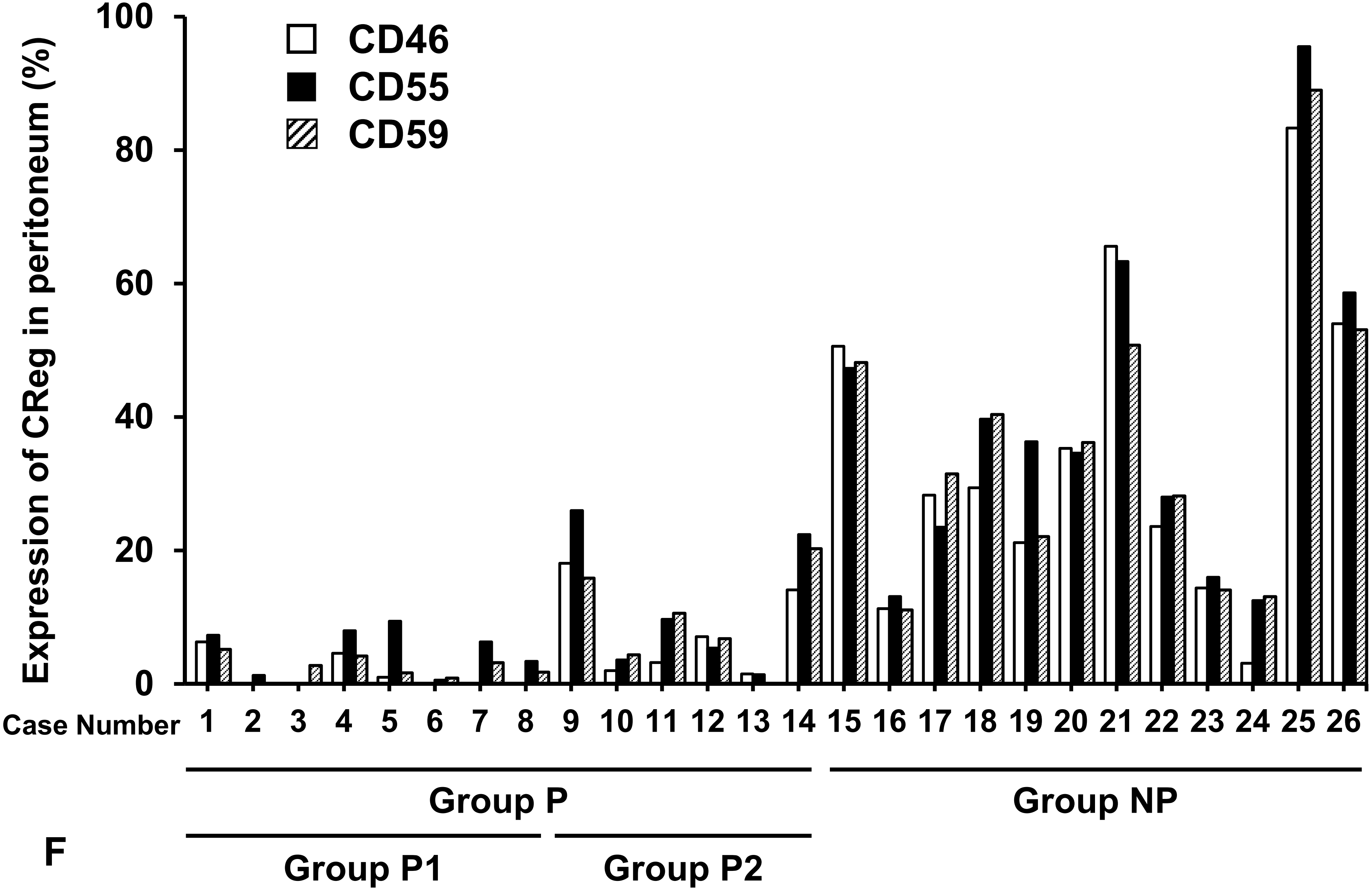
CD46
CD55
CD59
Expression of CReg in peritoneum (%)
100
80
60
40
20
0
Case Number
1 2 3 4 5 6 7 8 9 10 11 12 13 14 15 16 17 18 19 20 21 22 23 24 25 26
Group P
Group NP
Group P1
Group P2

F

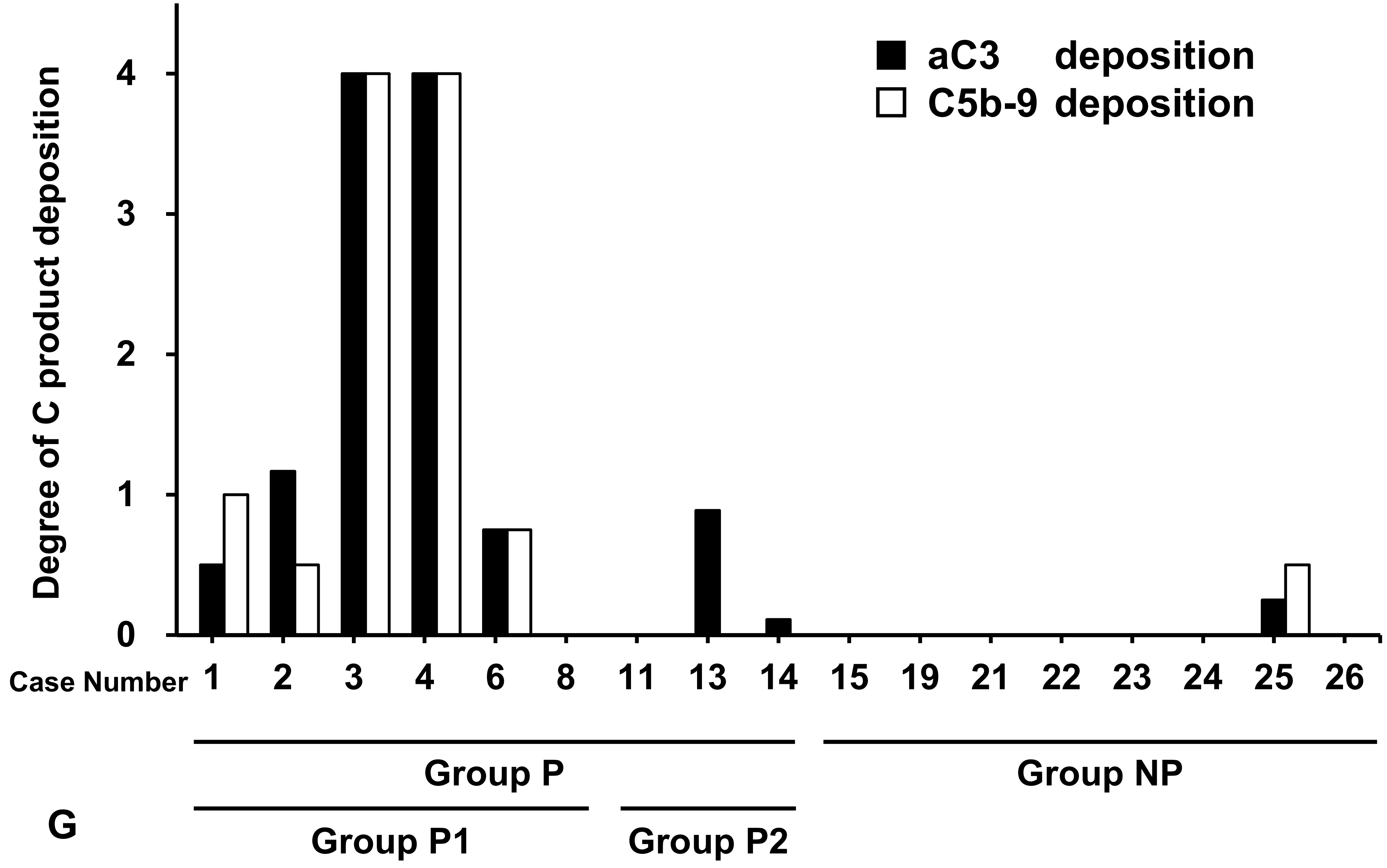
Degree of C product deposition
4
3
2
1
0
aC3 deposition
C5b-9 deposition
Case Number
1
2
3
4
6
8
11
13
14
15
19
21
22
23
24
25
26
Group P
Group NP
G
Group P1
Group P2

Supplementary Figure 1. Proportion of cytokeratin, accumulation of inflammatory cells, degree of fibrin and fibrosis, expression of membrane complement(C) regulators (CRegs) and complement activation products in each peritoneal biopsy sample evaluated in the present study. Graphs (**A–G**) show degree of cytokeratin-positive expression, number of esterase-positive cells, number of CD3-positive cells, number of CD68-positive cells, degree of fibrosis/fibrin deposition, expression of CRegs, and degree of C activation products in individual cases. Group P represents biopsy samples accompanied by PD-related peritonitis. Group NP represents samples without PD-related peritonitis as of the time of peritoneal biopsy. Group P is further divided into subgroups P1 and P2. Samples in Group P1 represent peritoneal biopsy samples of patients with *Candida* spp. or *P. aeruginosa* peritonitis. Samples in Group P2 represent those of patients with Gram-positive cocci. Numbers along the X-axis correspond to the Case numbers in Table 2.

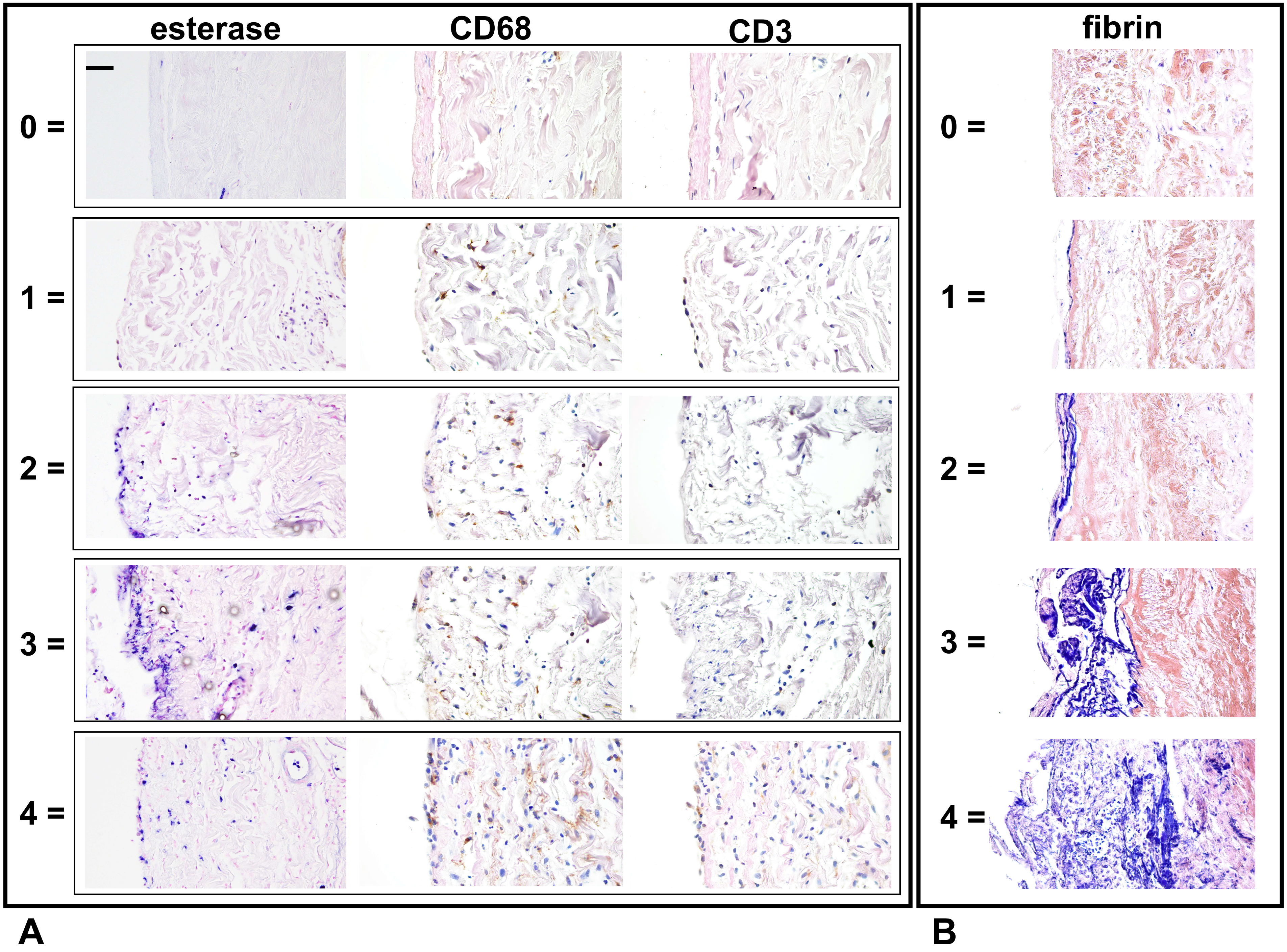

Tissue injury score [0-8] = total inflammatory cells score [0-4] + fibrin deposition score [0-4]

Supplementary Figure 2. Assessment of tissue injuries as tissue injury score. Calculation of tissue injury score was described in the text body. Briefly, to assess the score, our evaluated examples are shown with specific pictures of scores 0, 1, 2, 3, and 4 for cellular accumulations (combination of esterase-positive cells, CD68-positive cells and CD3) (**A**) and severity of fibrin deposition (**B**). We used tissue injury score as the summing scores. Original magnification of A and B is ×400. A scale bar of 50 μm is shown in the left upper corner of image on top left in A.
